# Exploiting Double Exchange Diels-Alder Cycloadditions for Immobilization of Peptide Nucleic Acids on Gold Nanoparticles

**DOI:** 10.3389/fchem.2020.00004

**Published:** 2020-01-23

**Authors:** Enrico Cadoni, Daniele Rosa-Gastaldo, Alex Manicardi, Fabrizio Mancin, Annemieke Madder

**Affiliations:** ^1^Organic and Biomimetic Chemistry Research Group, Department of Organic and Macromolecular Chemistry, Ghent University, Ghent, Belgium; ^2^Dipartimento di Scienze Chimiche, Università di Padova, Padova, Italy

**Keywords:** PNA, gold nanoparticles, AuNP, gold nanocluster, reversible diels-alder, controlled release, peptide nucleic acids

## Abstract

The generation of PNA-decorated gold nanoparticles (AuNPs) has revealed to be more difficult as compared to the generation of DNA-functionalized ones. The less polar nature of this artificial nucleic acid system and the associated tendency of the neutral poly-amidic backbone to aspecifically adsorb onto the gold surface rather than forming a covalent bond through gold-thiol interaction, combined with the low solubility of PNAs itself, form the main limiting factors in the functionalization of AuNP. Here, we provide a convenient methodology that allows to easily conjugate PNAs to AuNP. Positively charged PNAs containing a masked furan moiety were immobilized via a double exchange Diels-Alder cycloaddition onto masked maleimide-functionalized AuNPs in a one-pot fashion. Conjugated PNA strands retain their ability to selectively hybridize with target DNA strands. Moreover, the duplexes resulting from hybridization can be detached through a retro-Diels-Alder reaction, thus allowing straightforward catch-and-release of specific nucleic acid targets.

## Introduction

The use of DNA-decorated gold nanoparticles (AuNP) of various shape and size has been gradually established over the years, gaining a central role in the realization of a wide range of applications including DNA-based biosensing, gene delivery and *in vivo* imaging to name just a few examples (Ghosh et al., 2008; Homola, 2008; Boisselier and Astruc, 2009; Song et al., 2016). As a result, several methodologies for realizing DNA-AuNP complexes are available (Liu and Liu, 2017). On the other hand, alternative AuNP constructs with non-natural DNA mimics have not been explored so widely. The use of peptide nucleic acids (PNAs) can provide several advantages over the use of standard DNA, such as the formation of more stable and selective complexes with natural DNA and RNA targets which are less affected by experimental conditions (i.e., ionic strength, solvent polarity, presence of chaotropic agents, and pH) and improved resistance to chemical and biological degradation (Demidov et al., 1994; Nielsen, 2004). These characteristics endow PNAs with superior properties for the construction of AuNP based nucleic acid probes. AuNP-DNA assemblies typically require carefully tuned conditions to achieve hybridization with complementary strands, such as high ionic strength in order to overcome the high charge repulsion between the strongly negatively charged DNA nanoparticles and the complementary strands (Liu and Liu, 2017; Madsen and Gothelf, 2019). However, these high ionic strengths conditions, at the same time, can destabilize charged nanoparticles, causing them to aggregate (Behrens et al., 1998; Pamies et al., 2014).

Despite the aforementioned advantages, only few examples of well-described procedures for functionalization of AuNP with PNA are reported. One of the principal reasons is connected to the tendency of the neutral PNAs to randomly adsorb onto the gold surface, rendering a covalent functionalization through standard thiol-gold bond formation more difficult. In a first example from 2003, Chakrabarti et al. already reported on the difficulties encountered in conjugating standard PNAs to gold nanostructures (Chakrabarti and Klibanov, 2003). In fact, the use of uncapped N-terminal cysteine for the decoration of citrate-stabilized AuNPs, caused an immediate and irreversible aggregation as a consequence of the decreased surface negative charge density, but it was reported that this destabilization could be reduced by adding a poly-Glu tail at the C-terminus. Using this methodology, they were later able to synthesize PNA-decorated nanoparticles, but the functionality of PNA was compromised and no clear evidence of hybridization to complementary DNA was detected (Murphy et al., 2004). An effective approach for the preparation of fully functional PNA-decorated nanoparticles was finally reported by Duy et al., who employed the TWEEN-20 surfactant to prevent nanoparticle aggregation during the exchange between the citrate anions on the surface of the gold nanoparticles and thiolated PNA strands (Duy et al., 2010). In a more recent work a long chain, PEG-based, ω-mercapto carboxylic acid was used to create a monolayer providing both steric and electrostatic stabilization to the AuNPs. In a second step PNA strands, containing a variety of mono- and trithiol linkers conjugated at the N-terminus, could be covalently attached to the surface by thiol exchange in the presence of TWEEN-20 and a triphenyl phosphine as antioxidant, achieving the synthesis of stable PNA-AuNPs (Anstaett et al., 2013). The above examples suggest that the best strategy to obtain stable AuNP-PNA systems is the co-functionalization of the nanoparticle surface with stabilizing surfactants, either thiolated or not. Thiol exchange-based strategies, however, are difficult to control in terms of degree of substitution, and typically require large excesses of the entering species. In addition, special care is needed to avoid dimerization or oxidation of thiol-containing PNAs.

An alternative and elegant approach to overcome this problem is the use of click chemistry (Chen et al., 2017): either Huisgen 1,3-cycloadditions, or concerted reactions, such as Diels-Alder (DA) reactions, which have found numerous applications. In this context, bio-conjugation of nanomaterials, often exploits these approaches because of the tolerance of aqueous solutions and the high reaction selectivity (Sapsford et al., 2013). Diels-Alder-based approaches were previously exploited in NP context as a means to protect maleimide-containing thiols prior to their surface decoration, for the realization of NP-NP dimers, for biopolymer NP functionalization with antibodies and in stimuli-responsive drug release systems (Zhu et al., 2006; Liu et al., 2010; Ghiassian et al., 2015; Oluwasanmi et al., 2017).

**Figure 1:**
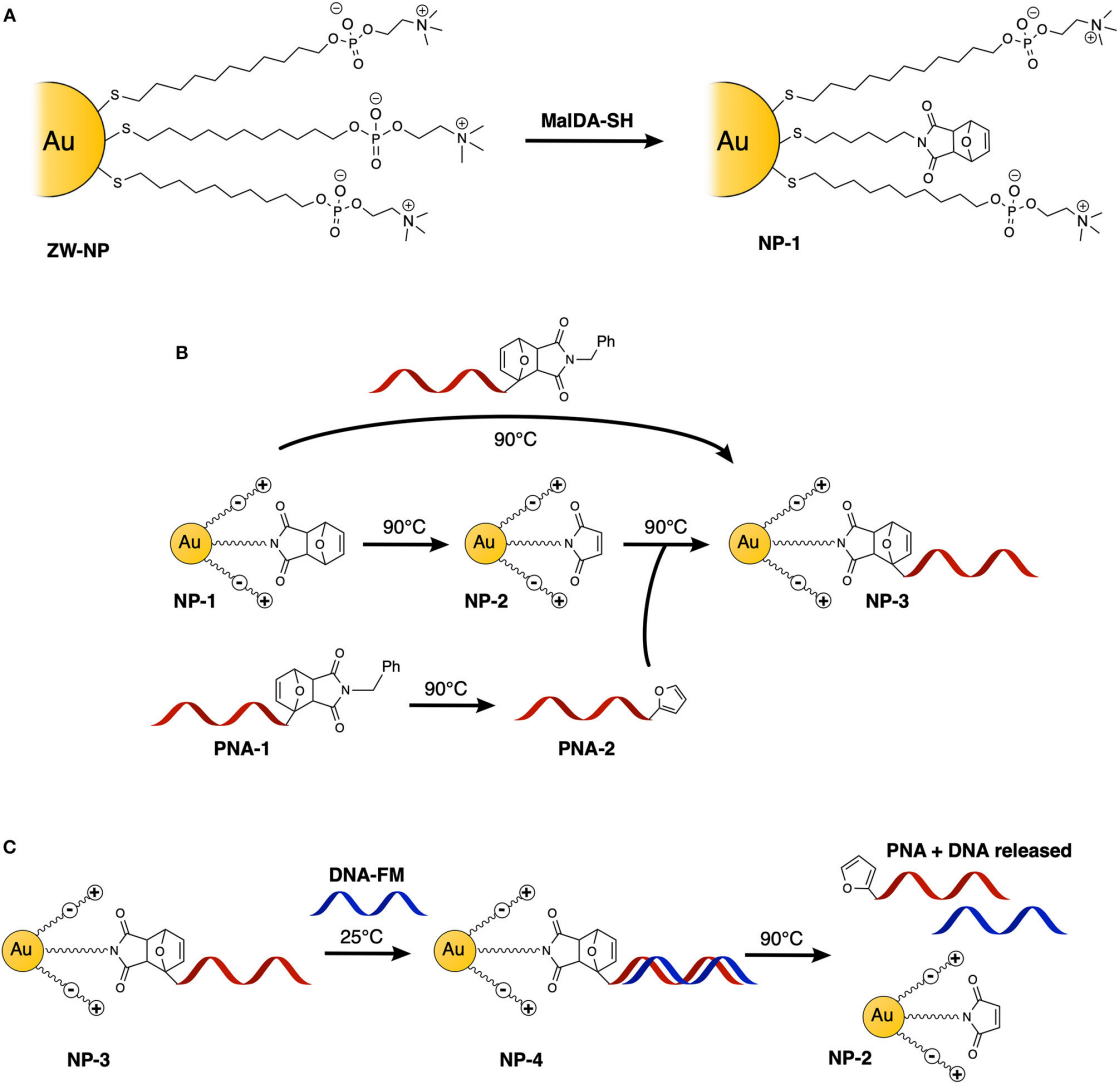
Schematic representation of the experiments performed in this study: **(A)** schematic representation of Diels-Alder protected maleimide containing nanoparticle **(NP-1)** synthesis starting from its precursor **ZW-NP**; **(B)** functionalization of AuNPs with PNA, via direct one-step double-exchange Diels-Alder and two-step Diels-Alder methodologies; **(C)** hybridization and release experiments with DNA.

In the context of our previous work on furan-oxidation based nucleic acid interstrand crosslinking (Manicardi et al., 2016), we recently developed a Diels-Alder/retro-Diels-Alder (DA/rDA) approach for the protection of furan moieties during their incorporation in PNA strands to avoid C5 alkylation of the aromatic ring during cleavage of the furan-modified PNAs from the solid support (Elskens et al., 2017). These studies have inspired us to exploit such furan-modified PNA strands for the realization of a simple methodology for AuNP functionalization (Figure 1). Here we report on the one-pot conjugation of a maleimide-protected furan-PNA to furan-protected maleimide-AuNPs and demonstrate the capability of this system to retain selective recognition of the target DNA as well as the possibility to release the resulting duplex system from the nanoparticle.

## Results and Discussion

### Synthesis of Gold Nanoparticles and Maleimide Linker

As evident on the basis of the examples discussed in the introduction, thiol protected nanoparticles have certain advantages over citrate stabilized ones. Indeed, the relatively strong covalent binding of thiol-based coating molecules and the steric stabilization they provide, guarantee better stability during the purification steps after PNA functionalization, compared to the weak binding and electrostatic stabilization provided by citrate anions. In addition, thiols grant the possibility to prepare overall neutral monolayers, which helps to minimize electrostatic interactions between the monolayer and PNA or DNA molecules. Finally, using thiols as coating agents it is possible to prepare ultrasmall (about 2 nm core size) nanoparticles. We chose this specific size for two reasons: first of all, by using 2 nm AuNPs it is possible to investigate in detail the composition of the monolayer by using solution NMR spectroscopy (in the case of larger nanoparticles the use of NMR spectroscopy is hampered by the massive signal broadening due to the slow tumbling). Secondly, the chosen size ensures the absence of a plasmonic band in the UV-Vis spectrum, that could limit the possibility for optical characterization of the AuNP-PNA complex.

**Figure 2:**
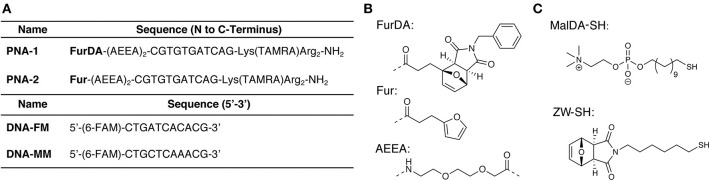
Sequences and small molecule building blocks used in this work. **(A)** PNA and complementary DNA sequences. **(B)** Structures of the monomers and spacers incorporated in the PNA sequences. **(C)** Thiols used for AuNP synthesis.

AuNPs were synthesized using a well-established two-phase two-step solution procedure (Manea et al., 2008). This involves the reduction of ${\text{AuCl}}_{4}^{-}$ in toluene (tetraoctylammonium bromide was used for phase transfer) by dioctylamine at first and then by NaBH_4_. The amount of dioctylamine present in the reaction mixture also determines the size of the gold core, since this molecule weakly binds to the particle surface. For this exploratory study, we chose to use 20 eq of dioctylamine to obtain AuNPs with an average gold core around 1.6 nm diameter (average formula Au_127_SR_38_). Dioctylamine-coated nanoparticles so obtained were then functionalized by the simple addition of the desired thiol. In this case, the nanoparticles were passivated with a zwitterionic thiol (**ZW-SH)** featuring a phosphorylcholine moiety (Figure 2C). We chose this thiol since it is well-known to give readily soluble and stable nanoparticles, and it has been already used to make biocompatible and bioconjugated nanoparticles with mixed monolayers (Jha et al., 2017). In order to be able to perform the PNA functionalization, a second thiol, bearing a furan-protected maleimide moiety (**MalDA-SH**, shown in Figure 2C), was inserted in the AuNPs via a thiol exchange reaction. The reaction simply required the addition of 0.5 eq of thiol (with respect to the overall amount of **ZW-SH**) to a solution of **ZW-NP** in methanol, followed by overnight stirring at 35°C. A 14% substitution of **ZW-SH** with **MalDA-SH** was estimated by ^1^H-NMR (**NP-1**, see Figure S11). The substitution achieved is optimal to guarantee the solubility of the nanoparticle and the coupling of the PNA. By changing the equivalents of thiol added, it is possible to tune the degree of substitution, however, when performing the exchange reaction with 1 eq of thiol (leading to a potential exchange of 25–30%), the resulting monolayer is too hydrophobic and the NPs were not completely soluble in water (data not shown). Finally, the possibility to free the maleimide group was demonstrated by heating the AuNPs at 90 °C for 16 h, affording **NP-2**. The deprotection can be followed by ^1^H-NMR spectroscopy: after 7 h a 50% conversion could be concluded from the spectrum, while after 24 h almost quantitative disappearance of **MalDA-SH** signal was noticed together with the appearance of unknown degradation products (see Figure S12). Therefore, an intermediate deprotection time was chosen, leading to a composition of the coating monolayer of **NP-2** containing both **Mal-SH** and a small amount of residual **MalDA-SH** (for the sake of simplicity in Figure 1A only the most abundant form is reported). However, this is not affecting the PNA coupling.

### PNA Synthesis

Although **ZW-SH** does not present a net charge, **NP-1** have a slightly positive ζ-potential (2.4 ± 0.9 mV). For this reason, two arginine residues were placed at the C-terminus of the PNA probes, which, besides guaranteeing improved PNA solubility, should allow to maintain the PNA tails oriented toward the solution (Biscaglia et al., 2017), and further favored DNA attraction via electrostatic interaction. Furthermore, a lysine residue carrying a 5-carboxytetramethylrhodamine (TAMRA) was included to facilitate monitoring of the nanoparticle functionalization. Finally, to enable Diels-Alder conjugation, a N-benzylmaleimide masked furan moiety was included in the N-terminal position following two 2-(2-(2-aminoethoxy)ethoxy)acetic acid (AEEA) spacers (**PNA-1**, see Figure 2A). The required **FurDA-OH** building block (structure shown in Figure 2B) could be conveniently synthesized in a single step by heating 2-furanpropionic acid in presence of an excess of maleimide in ethyl acetate (see Supplementary Material). The pure exo-adduct then precipitates from the solution driving the reaction to completion and allowing purification by simple filtration and re-crystallization.

In order to test the feasibility of the DA functionalization approach, a PNA bearing an unprotected furan (**PNA-2**) was also synthesized by removing the maleimide moiety of **PNA-1** under rDA conditions. Given the instability of the AEEA spacer when heated in the acidic solution resulting from the PNA cleavage, a new protocol for rDA under basic conditions was developed (see Supplementary Material), differing from rDA-based furan deprotection conditions previously reported (Elskens et al., 2017).

### Conjugation of the Probe via Diels-Alder Cycloaddition

**Figure 3:**
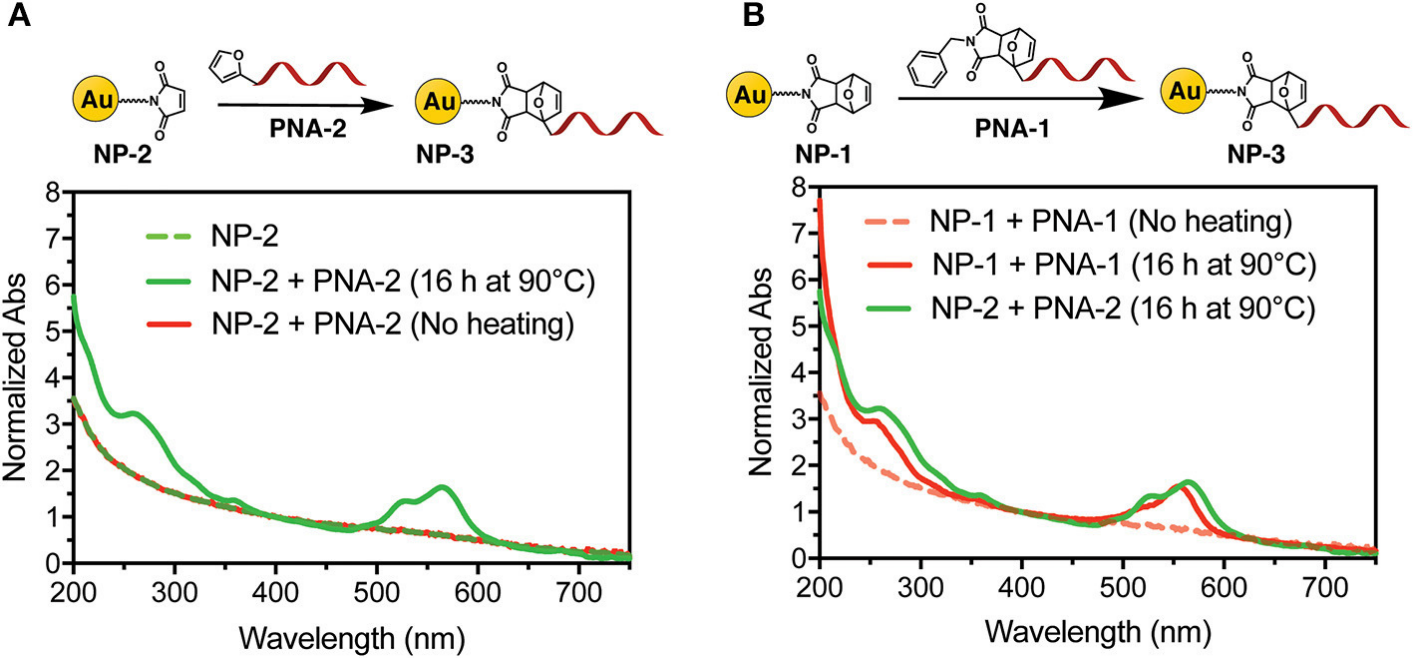
UV-Vis spectra of **NP-3** (normalized at 400 nm). **(A) NP-3** obtained via a two-step Diels-Alder approach; **(B)** comparison of **NP-3** obtained via the one-step double exchange Diels-Alder (red line) vs. the two-step approach (green line), after heating at 90°C for 16 h.

To first demonstrate the possibility to decorate AuNP with PNA using a DA approach, direct adduct formation between **NP-2** and **PNA-2** was tested. A buffered solution (PBS pH 7.4) of **NP-2** containing 50 μM maleimide and 100 μM **PNA-2** was heated at 90°C for 16 h. After purification by filtration on a 10 k Da cut-off filter the system was characterized by UV spectroscopy. As shown in Figure 3A the characteristic absorption of the PNA and of the TAMRA, 260 and 550 nm respectively, were clearly visible on top of the AuNP scattering, while they were absent in a similar experiment that was maintained at room temperature for the same time.

Next, we tested the possibility to uncage the required functional groups on **NP-1** and **PNA-1** and form the desired **NP-3** in a one-pot fashion. In this setup, formation of the DA adduct in **NP-3** is enthalpy driven by the higher stability of the adduct deriving from 2-alkyl furan as compared to those deriving from furan (Boutelle and Northrop, 2011), while, at the same time, the adduct formed by the two other counterparts should show reduced solubility in aqueous media and precipitate.

Interestingly, the absorption spectrum of **NP-3** obtained via double exchange DA reaction does not shown significant differences as compared with the same product deriving from the two-step DA approach. To validate the results, a negative control experiment was performed without heating the solution, and the resulting UV spectrum lacks the characteristic absorption peaks of the PNA (Figure 3B). Finally, once the feasibility of the methodology was established, we focused our attention on the kinetics of the functionalization process. As shown in Figure S1, after 2 h of heating, the conjugation with the PNA appears to have reached half of the maximum value, which is observed after 4 h.

### DNA Hybridization and Release

**Figure 4:**
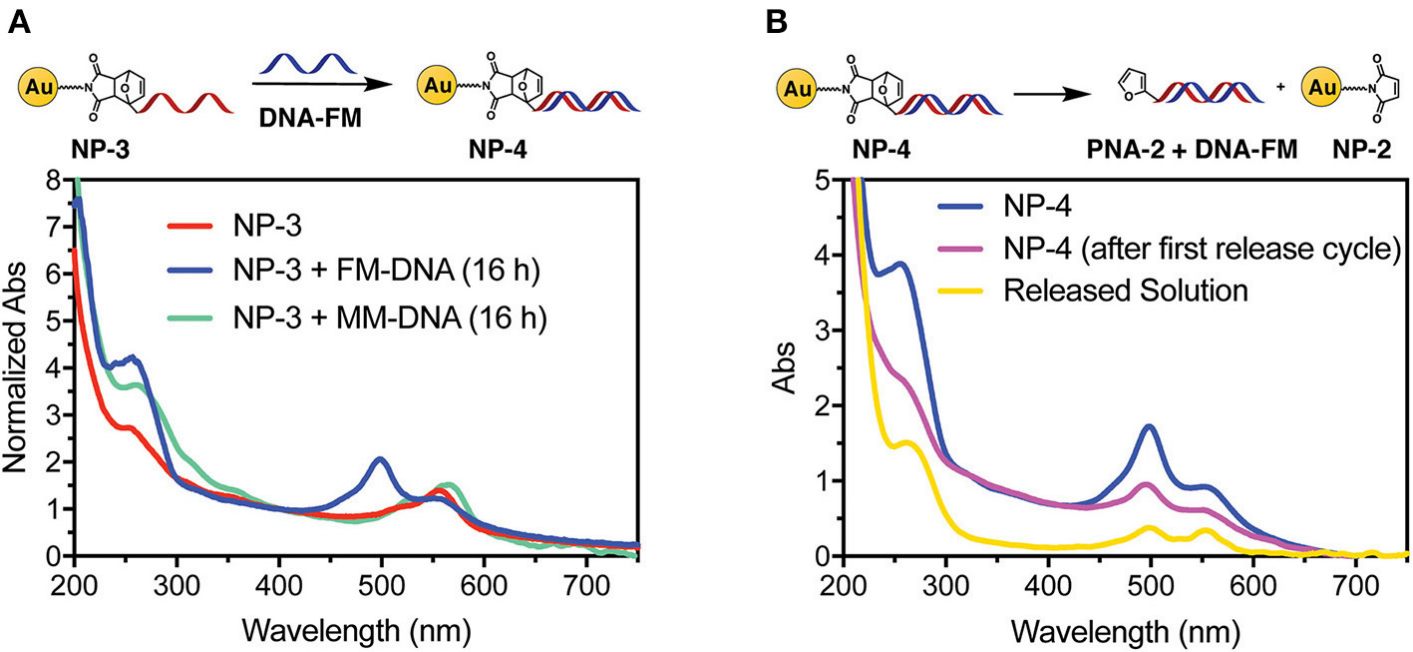
UV-vis spectra of **NP-4** after hybridization with target DNA **(A)** and after thermal release **(B)**.

In order to confirm the ability of the attached PNA probe to be radially exposed to the solution and thus maintain its ability to recognize target sequences, hybridization experiments were performed. For this purpose, a 5′-fluorescein (FAM) labeled DNA designed to be complementary to the PNA sequence was employed (see Figure 2A for the DNA sequences). UV analysis of **NP-4** obtained after overnight hybridization with **DNA-FM** in PBS pH 7.4 clearly shows the presence of the characteristic absorption of the FAM peak at 490 nm, as shown in Figure 4A.

To exclude the possibility of non-specific adsorption of the negatively charged DNA to the positively charged **NP-3** we performed overnight hybridization experiments in presence of the mismatched **DNA-MM** sequence. In this case the FAM peak was not present in the UV spectrum of the purified sample, thus confirming the selectivity of the hybridization (Figure 4A). Similar UV profiles were also obtained after 1-h hybridization See Figure S2, indicating that the time allowed for hybridization under these conditions can be shortened.

As a final step, we also wanted to show the possibility to thermally release the attached probes from the AuNP. A **NP-4** solution was therefore heated overnight at 90°C to release the duplex via rDA, then quickly cooled in an ice bath, and filtered through a 10 kDa cut-off filter. As shown in Figure 4B the released solution (yellow curve) clearly shows both FAM (490 nm) and TAMRA (550 nm) components. Although the DNA release from the system could also result from melting the PNA:DNA complex, the presence of the TAMRA peak in the filtered solution is a clear evidence of the release of the PNA probe from the AuNP. MALDI-TOF analysis of the filtrate also confirmed this hypothesis (see Figure S4). A second rDA treatment was carried out to achieve an almost complete release from the AuNP surface. However, in addition to the PNA release, aggregation of the AuNPs was suspected from the observation of a broad band around 520 nm (see Figure S3B).

## Materials and Methods

### Retro Diels-Alder on AuNP

**NP-2** are prepared by heating the desired volume (typical 1 mL, 500 μM concentration in maleimide linkers) of an aqueous **NP-1** solution for 16 h at 95 °C in a glass vial. The solvent is then removed under reduced pressure to remove the furan. The nanoparticles are finally re-suspended in the initial volume of water.

### PNA-AuNP Conjugation

In a typical experiment, 100 μL of a PBS solution (pH 7.4) containing PNA (50 μM) and AuNP (concentration adjusted to provide 25 μM maleimide-containing linker) is heated for 4 h at 90°C. This solution is then diluted with 20 μL of PBS buffer and purified by filtration over Amicon 10 kDa ultracentrifuge filters (14000 rcf, 5 min). The remaining solution is re-diluted by adding 200 μL of PBS and filtrated again to remove impurities (unreacted PNA, DA byproducts). The concentrated solution of **NP-3** is then recovered and analyzed via UV spectrometry.

### Hybridization Experiments

In a typical hybridization experiment, a 100 μL PBS solution (pH 7.4) containing **NP-3** at 10 μM and 5′-FAM-labeled DNA at 20 μM, was allowed to hybridize under shaking for at least 1 h at 25°C. The hybridized solution was then diluted to 200 μL with PBS and purified by filtration over Amicon 10 kDa ultracentrifuge filters (14000 rcf, 10 min). The remaining solution was washed twice by adding 200 μL of PBS and filtrated again to remove impurities (unhybridized DNA). The concentrated solution of **NP-4** was finally recovered and analyzed via UV spectrometry.

### Release Experiments

In a typical release experiment, 50 μL of **NP-4** solution obtained from the previous step were placed in an Eppendorf tube, shaken overnight at 90°C. The solution was thereafter placed in an ice-cold bath for 10 min (to avoid re-attachment) and purified by filtration over Amicon 10 kDa ultracentrifuge filters (14000 rcf, 10 min). The obtained filtrate was analyzed via UV before washing. Afterwards, the nanoparticles were washed adopting the same procedure of the previous paragraph. The final concentrated nanoparticle solution was finally recovered and analyzed via UV spectrometry.

## Conclusions

In conclusion, we reported on a convenient methodology that allows conjugating peptide nucleic acids to gold nanoparticles, exploiting the reversibility of the Diels-Alder cycloaddition. This conjugation approach permits to overcome the problems related to the use of thiolated PNAs, making synthesis, handling, and purification of the probes easier, increasing the yields, and prolonging their shelf-life. In principle, varying the maleimide content of the precursor nanoparticle, allows modifying the PNA content without employing a large excess of PNA probe. The possibility to perform this functionalization via a one-pot double exchange Diels Alder protocol without affecting the outcome of the reaction further expands the applicability of this methodology, extending the shelf-life of the nanoparticle system given the increased stability of the protected maleimide **NP-1** as compared to its unprotected version **NP-2**. The simplicity of the protocol together with the robustness of the reactive moieties will also permit to expand this methodology to the functionalization of other nanosystems or surfaces. In addition, we demonstrated the ability of the attached system to retain its recognition abilities toward target DNA sequences as well as the possibility to use this type of connection for the realization of catch-and-release systems. We strongly believe that further optimization of the final release step will permit the application of this methodology to stimuli-responsive nanosystems.

## Data Availability Statement

All datasets generated for this study are included in the article/Supplementary Material.

## Author Contributions

EC, AMan, Amad, and FM designed the project and the experiments. AMan, AMad, and FM supervised the project overall. EC and AMan performed the synthesis of the PNA probes and building blocks, DR-G and EC synthesized the nanoparticles and thiolated linkers. The manuscript was prepared by EC, DR-G, and AMan and revised by all the authors.

### Conflict of Interest

The authors declare that the research was conducted in the absence of any commercial or financial relationships that could be construed as a potential conflict of interest.
