# Supplementary material for: Exploiting Double Exchange Diels-Alder Cycloadditions for Immobilization of Peptide Nucleic Acids on Gold Nanoparticles
Source: Front Chem. 2020 Jan 23;8:4. doi: 10.3389/fchem.2020.00004 (PMC6989547; doi:10.3389/fchem.2020.00004)
Supplement: Supplementary file 1 [file Table_1.pdf]

Supplementary Material

Exploiting double exchange Diels-Alder Cycloadditions for immobilization of Peptide Nucleic Acids on Gold Nanoparticles

Enrico Cadoni[1,2], Daniele Rosa-Gastaldo[2], Alex Manicardi[1], Fabrizio Mancin[2], Annemieke Madder*[1]

[1] Organic and Biomimetic Chemistry Chemistry Research Group, Department of Organic and Macromolecular Chemistry, Ghent University, Krijgslaan 281 S4, B-9000 Ghent, Belgium

[2] Dipartimento di Scienze Chimiche, Università di Padova, Via Marzolo 1, Padova, 35131, Italy

1 General *2*

2 Synthesis *3*

2.1 Monomer and linker synthesis 3

2.2 PNA synthesis 3

2.3 Nanoparticle Synthesis 4

3 UV-Vis Analysis *5*

3.1 Double exchange optimization 5

3.2 Hybridization experiment optimization 5

3.3 Release experiments 6

4 Characterizations *8*

4.1 FurDA-OH characterization 8

4.2 PNA Characterization 9

4.3 Nanoparticles characterization 10

5 Supplementary references *15*

1 General

All reagents were purchased from Sigma-Aldrich, Merck, Fluorochem and used without further purification. Dry DMF was stored over 4 Å molecular sieves. TLCs were run on Merck silica 60 on aluminum sheets. Column chromatography was performed as flash chromatography on Grace silica 60 (0.060- 0.200 mm). DNA sequences were purchased from IDT (Leuven, Belgium).

NMR spectra were recorded on a Brucker Advance 300, 400 or 500. δ values are expressed in ppm relative to the residual solvent peak. The following abbreviations are used to explain the multiplicities: s=singlet, d=doublet, t=triplet, q=quadruplet, m=multiplet, and br=broad.

HPLC-MS data were collected on an Agilent 1100 Agilent Technologies, Machelen, Belgium Series instrument with a Phenomenex Kinetex C18 100 Å column (150×4.6 mm, 5 µm at 35°C) connected to an ESMSD type VL mass detector (quadrupole ion trap mass spectrometer, Agilent Technologies, Machelen, Belgium). **HPLC1**: (A) 0.1% HCOOH in H_2O and (B) MeCN; 100% A for 2 min, then a gradient from 0 to 100% (B) over 6 min was used, followed by 2 min of flushing with 100% B at 1.5 mL/min.

HPLC purification of PNA oligomers was performed on an Agilent 1100 Series equipped with a Luna C18(2) (5 µm, 100 Å, 250x4.6 mm). (A) 0.1% TFA in H_2O and (B) MeCN; 100% (**HPLC2**, 100% A for 5 min, then a gradient from 0 to 50% B over 30 min at a flow rate of 4.0 ml/min).

UV-VIS spectra were recorded using a Thermofischer NanoDrop UV/VIS droplet reader.

DA Conjugation and release experiments were performed in 1.5 mL Eppendorf tube, the temperature was controlled using Eppendorf® Thermomixer Comfort. The solutions were filtered using Amicon ® Ultra-0.5 Centrifugal Filters (500 µL, 10kDa Molecular Weight Cut-Off), in an Eppendorf MiniSpin Plus centrifuge (rotor: F-45-12-11).

MALDI-TOF analysis was performed using an Applied Biosystems - 4800 Plus MALDI TOF/TOF™ Analyzer. As matrix, 100 mg/mL 2,5-Dihydroxybenzoic acid (DHB) in mQ water : MeCN (1:2) + 0.1% TFA was used.

TEM images were recorded on a Jeol 300 PX electron microscope. One drop of 1 mg/ml solution of AuNP was placed on the sample grid and removed gently using filter paper. TEM images were analysed with ImageJ software to measure the diameters distribution and the average formula.

TGA was run on 0.68 mg AuNP sample using a Q5000 IR model TA instrument under continuous air flow.

2 Synthesis

2.1 Monomer and linker synthesis

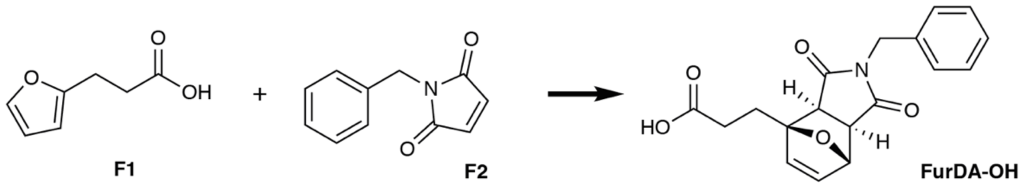

Scheme S1. Synthesis of **FurDA-OH** building block.

Synthesis of 3-(2-benzyl-1,3-dioxo-1,2,3,3a,7,7a-hexahydro-4H-4,7-epoxyisoindol-4-yl)propanoic acid [**FurDA-OH**]**:** in a sealed tube provided with a stirring bar 3-furan-2-propionic acid (1.635 g, 11.67 mmol, 1 eq.) is solubilized in 5 mL of AcOEt. 1-Benzyl-1*H*-pyrrole-2,5-dione (2.84 g, 15.17 mmol, 1.3 eq.) is then added and the mixture is heated at 70°C for 3 hours. Further amounts of maleimide are added over time up to 2 eq. The reaction is then diluted with 70 mL of hexane and left triturating overnight. The solid is then reprecipitated twice from AcOEt/hexane to obtain **FurDA-OH** as brownish solid (2.869 g, 75.2%). **TLC (AcOEt):** Rf: 0.35; **^{1}H NMR (300 MHz, DMSO-d_6)** δ(ppm): 12.16 (s, 1H), 7.35 – 7.15 (m, 5H), 6.58 (dd, *J* = 5.7, 1.8 Hz, 1H), 6.49 (d, *J* = 5.7 Hz, 1H), 5.11 (d, *J* = 1.8 Hz, 1H), 4.56 (s, 2H), 3.13 (d, *J* = 6.4 Hz, 1H), 2.94 (d, *J* = 6.4 Hz, 1H), 2.45 – 2.31 (m, 2H), 2.28 – 1.98 (m, 2H); **^{13}C NMR (75 MHz, DMSO-d_6) δ(ppm):** 176.1, 174.8, 173.8, 138.2, 137.3, 135.9, 128.4, 127.2, 126.8, 90.4, 80.0, 50.2, 48.7, 41.2, 29.6, 24.7; **LC-MS (MeOH, HPLC 1)** Rt: 3.4 min; *m/z* calcd for $C_{18}H_{17}NO_5$ [M]: 327,11067, found:326.1 [M-H]$^-$, 653.1 [2M-H]$^-$; **HR-MS (MeOH)** m/z found 326.1020 [$C_{18}H_{16}NO_5$]$^-$.

Previously reported procedures were used for the synthesis of **ZW-SH** (Holmlin et al., 2001) and **MalDA-SH** (Huang et al., 2017). The characterizations of the final molecules matched the ones reported therein.

2.2 PNA synthesis

PNA synthesis is performed with standard manual Fmoc-based solid-phase synthesis using HBTU/DIPEA as activating mixture, using commercially available Fmoc-PNA-OH monomers (Biosearch Technologies, Bellshill). Rinkamide-ChemMatrix resin is first loaded with Fmoc-Arg$_{(Pbf)}$-OH as first monomer (0.2 mmol/g loading). Modification of lysine side chain with 5-TAMRA was performed after the elongation of the sequence and after Mtt deprotection using a 0.5% BtOH·H_2O solution in HFIP/DCM (3 minutes, 4 cycles) (Chouikhi et al., 2012). PNA cleavage was performed using a TFA:m-cresol (9:1) cleavage cocktail. After HPLC purification, the purity and identity of the PNAs were evaluated by LC-MS.

PNA Retro Diels-Alder protocol: the crude PNA obtained after cleavage is solubilized in mQ water at a final PNA concentration of 1 mM at pH ~11.5. The solution is then aliquoted in 1.5 mL Eppendorf and heated at 90°C for 2 hours.

Table S1. PNA sequences used in this study.

Name	Sequence (N to C terminus)	MW	ε ($M^{-1}cm^{-1}$)
**PNA-1**	Fur-$(AEEA)_2$-CGTGTGATCAG-Lys(TAMRA)Arg_2-NH_2	4476.57	145180
**PNA-2**	FurDA-$(AEEA)_2$-CGTGTGATCAG-Lys(TAMRA)Arg_2-NH_2	4299.37	145180

PNA-1: 13.2%; t_r: 3.62 min (HPLC1); ε = 145˙180 $M^{-1}cm^{-1}$; ESI-MS: *m/z* calcd 4486.57 [M]: 1496.1 $[MH_3]^{3+}$, 1122.3 $[MH_4]^{4+}$, 898.0 $[MH_5]^{5+}$, 748.5 $[MH_6]^{6+}$. **PNA-2**: 1.8 %; t_r: 4.42 min (HPLC1); ε = 145˙180 $M^{-1}cm^{-1}$; ESI-MS: *m/z* calcd 4299.37 [M]: 1434.3 $[MH_3]^{3+}$, 1075.3 $[MH_4]^{4+}$, 860.5 $[MH_5]^{5+}$, 717.3 $[MH_6]^{6+}$.

2.3 Nanoparticle Synthesis

Synthesis of AuNPs is performed following a two-step procedure as previously reported (Manea et al., 2008): a $HAuCl_4{\cdot}3H_2O$ (100 mg, 0.254 mmol, 1 eq) solution in water (1 mL) is extracted with a solution of tetraoctylammonium bromide (0.350 g, 0.636 mmol, 2.5 eq) in N_2 purged toluene (250 mL). After 15 minutes, dioctylamine (1.226 g, 5.078 mmol, 20 eq) is added to the resulting dark orange solution and stirred under N_2 for 1.5 hours until the solution appears colorless. The solution is then cooled down to 0°C in an ice bath and $NaBH_4$ (96.0 mg, 2.538 mmol, 10 eq) dissolved in H_2O (2 mL) is rapidly added. The solution turns black and it is left stirring in the same conditions for 90 minutes. The aqueous layer is removed and the desired thiol (0.508 mmol, 2 eq) dissolved in MeOH (2 mL) is rapidly added and the solution is further stirred for 3 hours. The AuNPs precipitate from the toluene solution, they are collected by centrifugation (5000 rpm, 5 min) and purified by subsequent dissolution (MeOH, 2 mL), sonication, and precipitation (2x 40 mL toluene, 2x 40 mL Et_2O). AuNPs are further purified by gel permeation chromatography on Sephadex LH-20 using methanol as eluent.

AuNP Retro Diels-Alder protocol: **NP-2** are prepared by heating the desired volume (typical 1 mL, 500 µM concentration in maleimide linkers) of an aqueous **NP-1** solution for 16 hours at 95 °C in a glass vial. The solvent is then removed under reduce pressure to remove the furan. The nanoparticles are finally re-suspended in the initial volume of water.

3 UV-Vis Analysis

3.1 Double exchange optimization

A 100 µL PBS solution (pH 7.4, 100 mM NaCl, 10 mM phosphate) containing **PNA-1** at 50 µM and **NP-1** at 25 µM concentration in maleimide-containing linker is heated for 2, 4 or 16 hours at 90°C. These solutions are then diluted with 200 µL of PBS and purified over 10kDa cut-off ultracentrifuge filters (5 minutes, 14000 rfc). The remaining solutions are washed by adding 200 µL of PBS and purified again. The concentrated solutions are then recovered and analyzed via UV spectrometry.

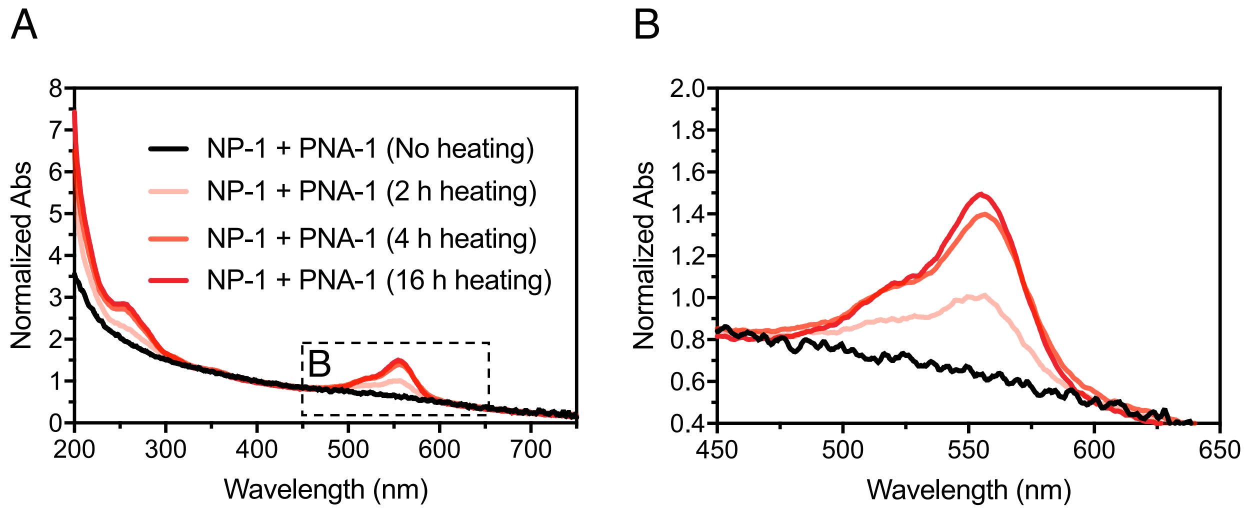

Figure S1: UV-Vis spectra of **NP-3** obtained after heating at various time points. A negative control was provided by hybridizing the solution at 20°C for 16 hours (continuous black line). **(A)** Full UV-Vis spectra (200-750 nm); **(B)** Zoom of the 450-650 nm region.

3.2 Hybridization experiment optimization

A 100 µL PBS solution (pH 7.4) containing 10 µM **NP-3** and 20 µM **DNA-FM** is equilibrated for 1, 2, 4 or 16 hours at 25°C under shaking. The hybridized solutions are then diluted to 200 µL with PBS (pH 7.4, 100 mM NaCl, 10 mM phosphate) and purified over Amicon 10k ultracentrifuge filters (5 minutes, 14000 rfc). The remaining solutions are washed twice by adding 200 µL of PBS and filtrated again. The concentrated solutions are then recovered and analyzed via UV spectrometry.

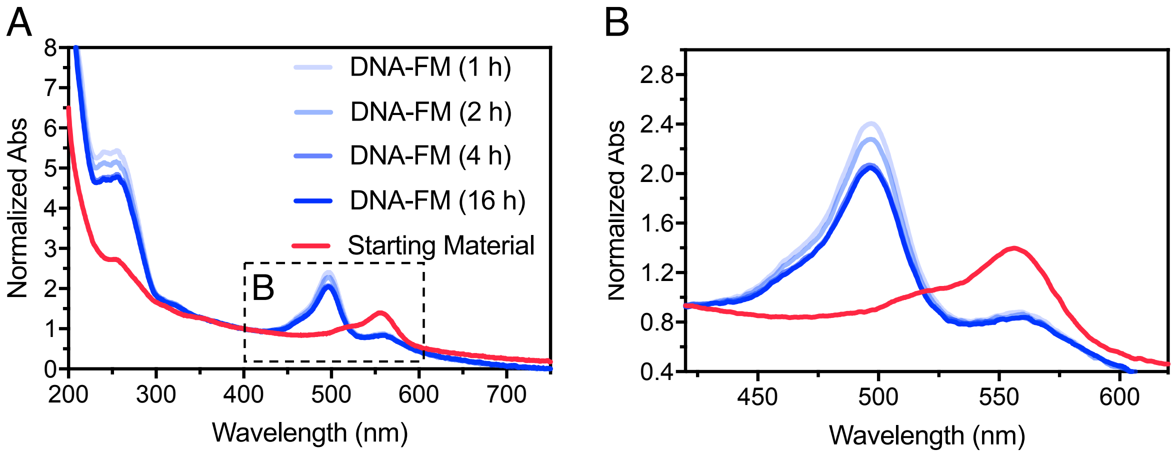

Figure S2. UV-Vis spectra of **NP-4**, after hybridization with **DNA-FM** for increasing time. **(A)** Full UV-Vis spectra (200-750 nm); **(B)** Zoom of the 420-620 nm region.

3.3 Release experiments

A 50 µL of **NP-4** solution obtained in the previous paragraph, is diluted to 200 µL, placed in a Eppendorf tube and shaken overnight at 90°C. The solution is thereafter placed in an ice-cold bath in order to minimize the re-assembly of the system and, once cooled down, immediately filtered over a 10k Da cut-off filter (10 minutes, 14000 rfc). The filtered solution is analyzed via UV and MALDI-TOF before washing. AuNP are washed using aforementioned washing protocol. A second cycle was repeated to ensure release of the PNA from the AuNP.

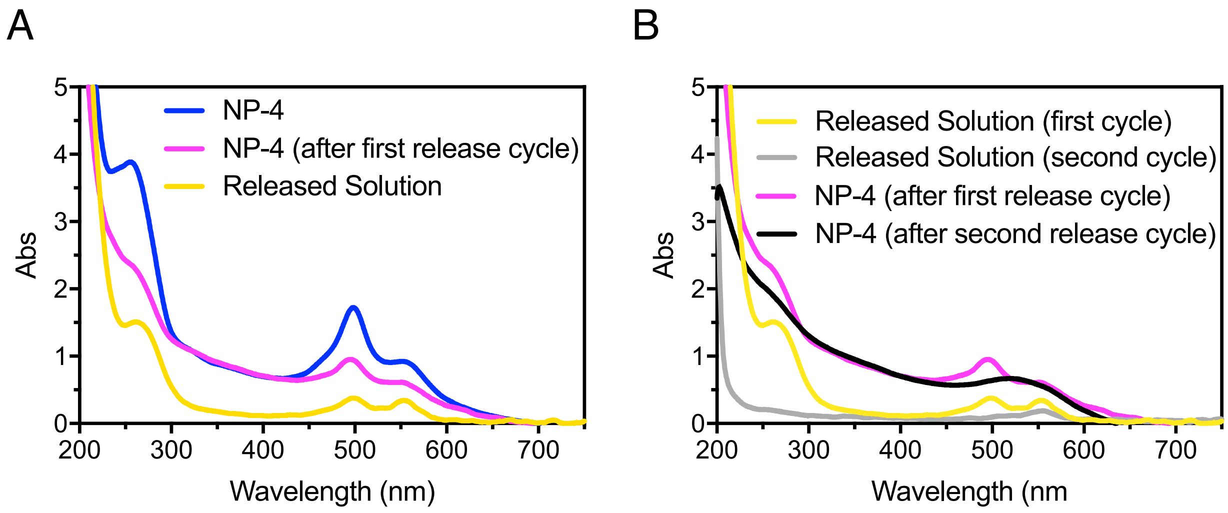

Figure S3. UV-Vis spectra of **NP-4** after rDA mediated release of the PNA:DNA complex **(A)**. Comparison of the two release cycles from **NP-4 (B).**

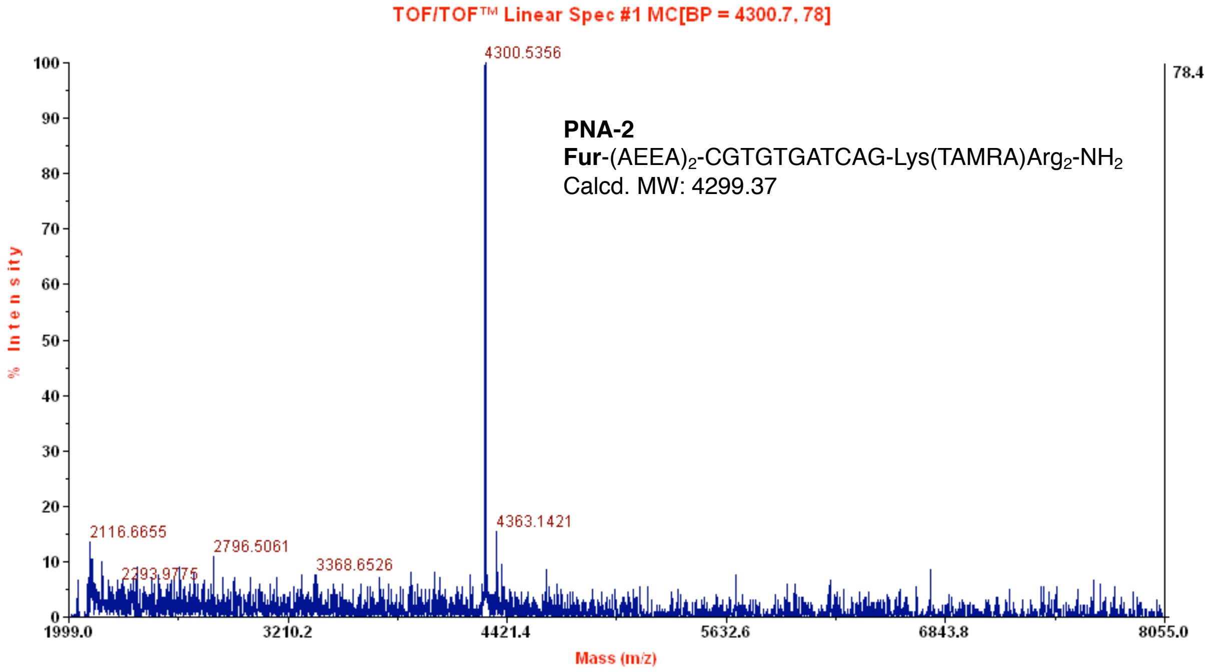

Figure S4. MALDI-TOF analysis of the filtrate, showing the presence of **PNA-2**. **(**Structure and MW reported).

4 Characterizations

4.1 FurDA-OH characterization

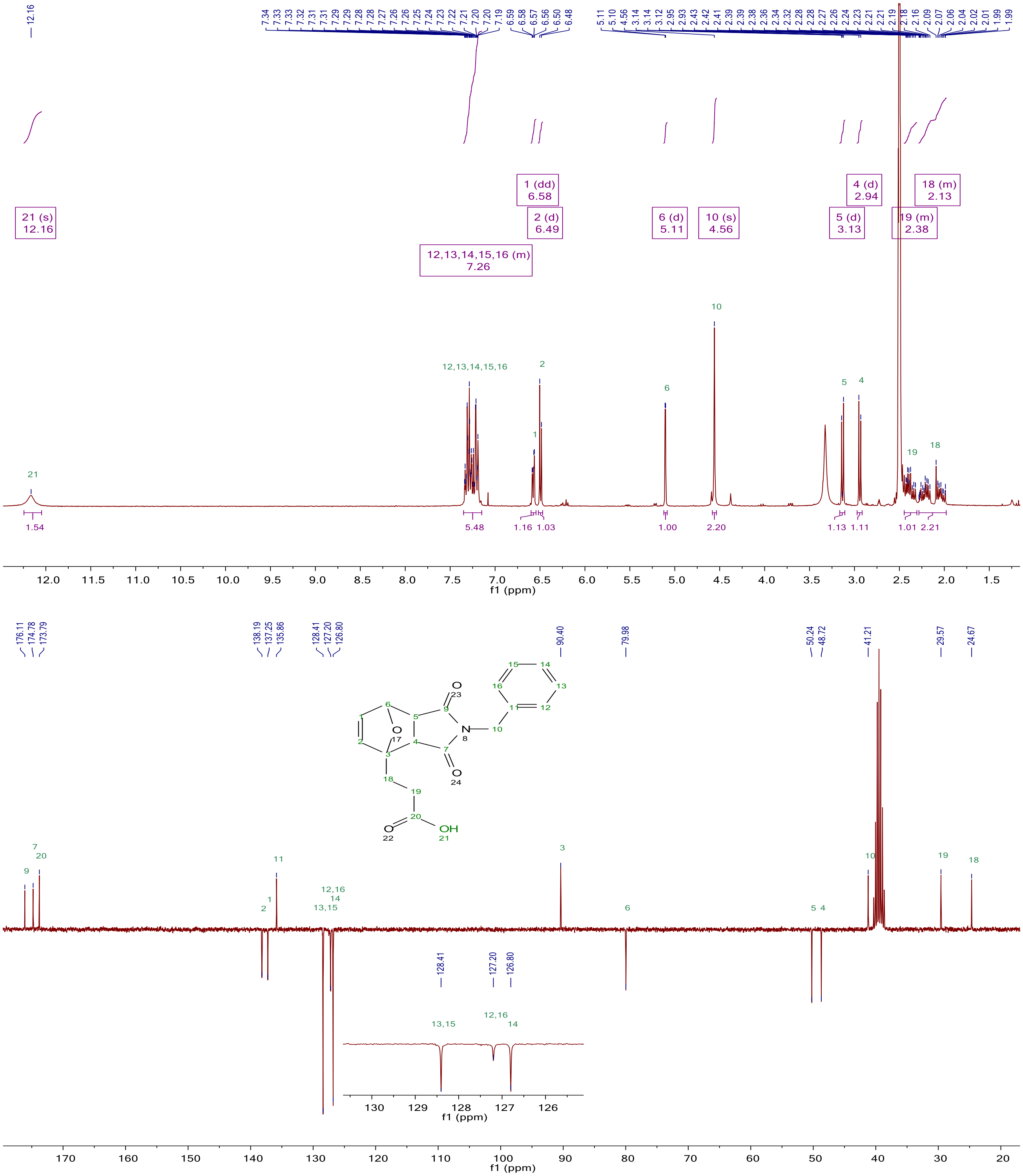

Figure S5. ^{1}H-NMR (300 MHz, top) and ^{13}C-NMR (75 MHz, bottom) spectra of **FurDA-OH** in DMSO-d^{6}.

4.2 PNA Characterization

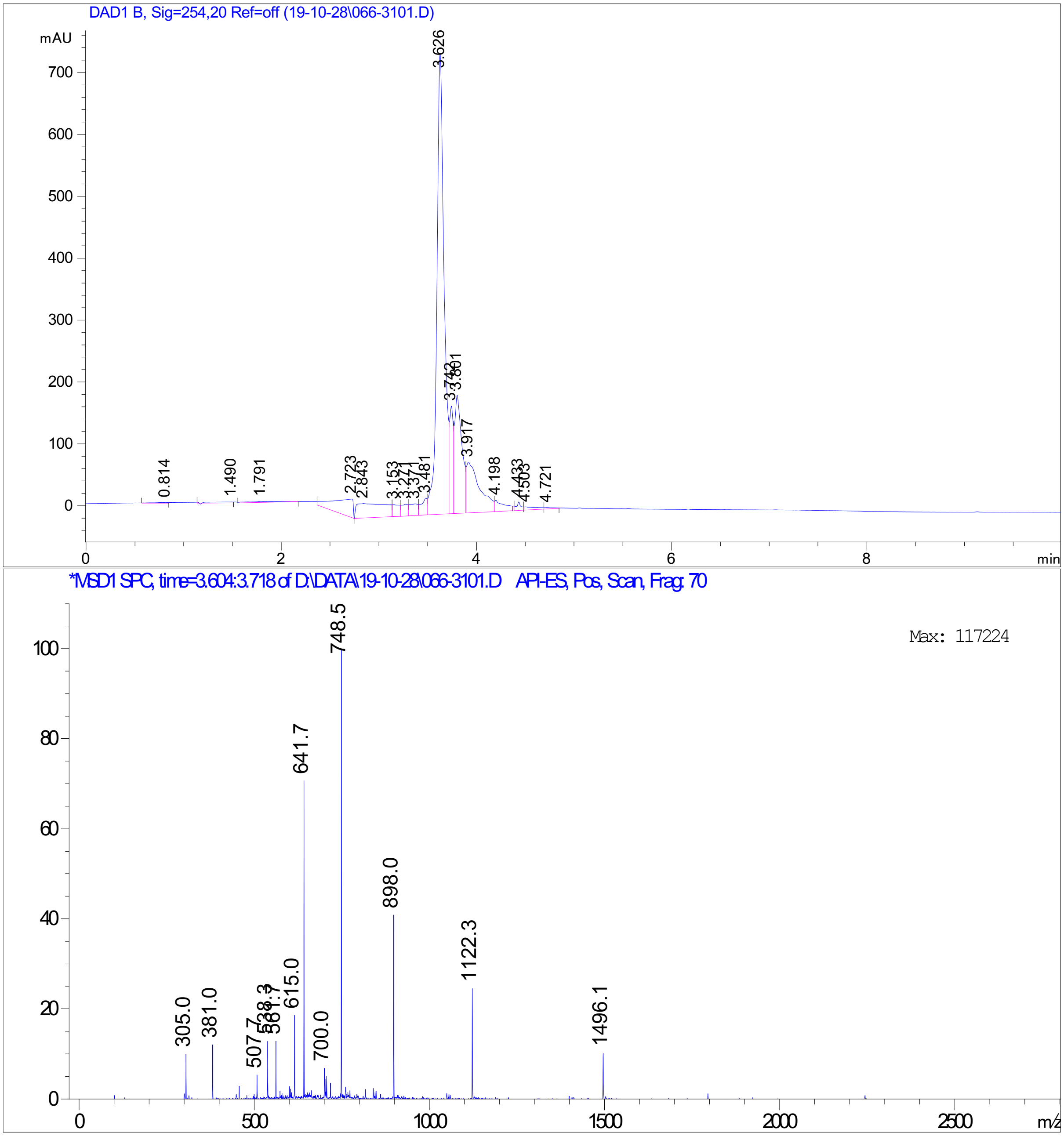

Figure S6. HPLC-MS analysis of the purified **PNA-1**. On top, HPLC-UV trace at 254 nm. At the bottom, MS spectrum of the corresponding peak at 3.62 min. Calcd MW: 4486.57

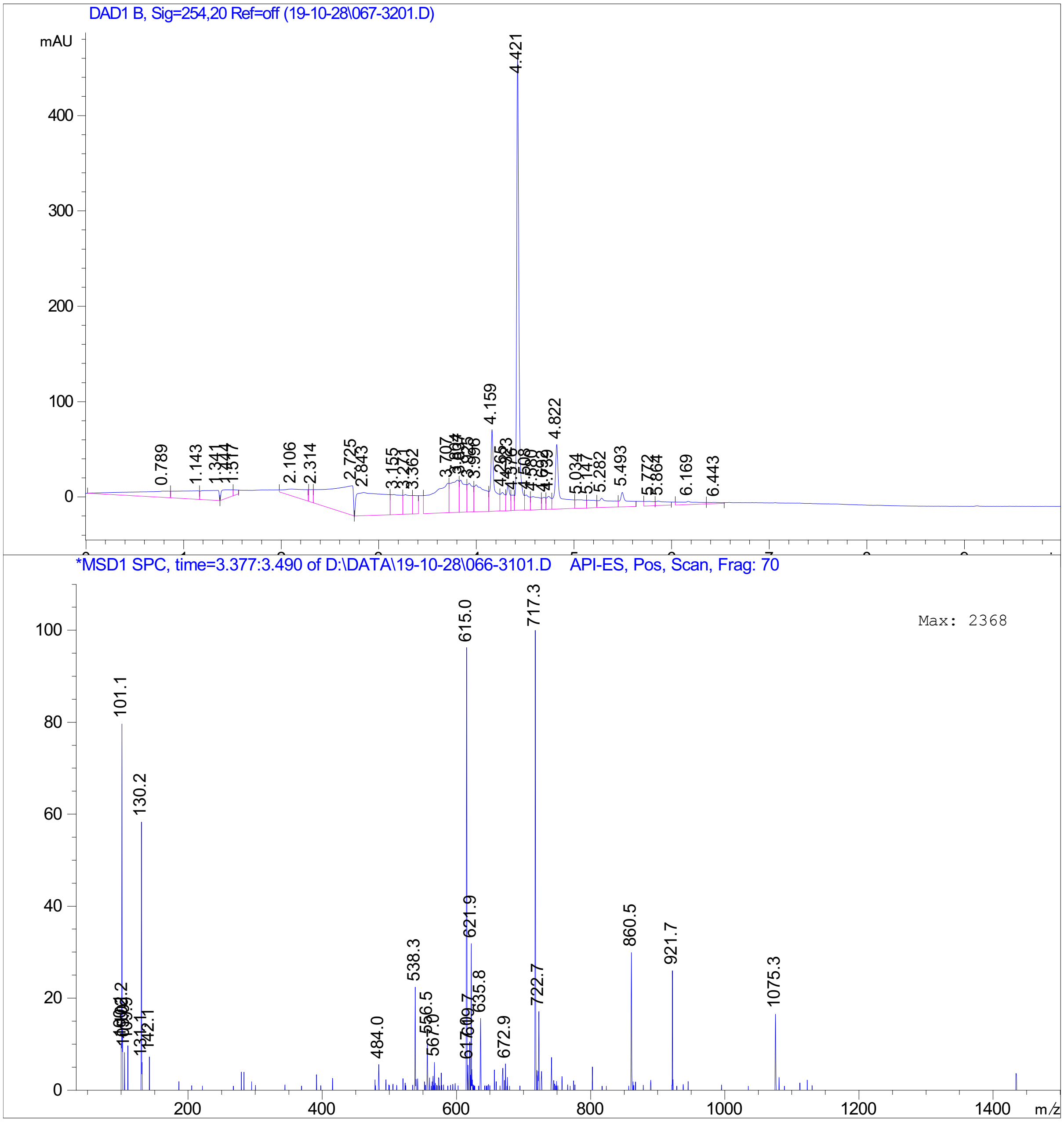

Figure S7. HPLC-MS analysis of the purified **PNA-2**. On top, HPLC-UV trace at 254 nm. At the bottom, MS spectrum of the corresponding peak at 4.42 minutes. Calcd MW 4299.37.

4.3 Nanoparticle characterization

The average diameter of the gold core for **ZW-AuNP**, as obtained from TEM analysis (Figure S8), is 1.6±0.3 nm. Using a spherical approximation of the gold and considering the weight loss in TGA (Figure S9) due only to the thiol loss, the average formula for **ZW-AuNP** is $Au_{127}SR_{38}$. The diffusion filter spectra in Figure S10 confirm that the thiols are grafted to the gold core. No relevant organic impurities are present.

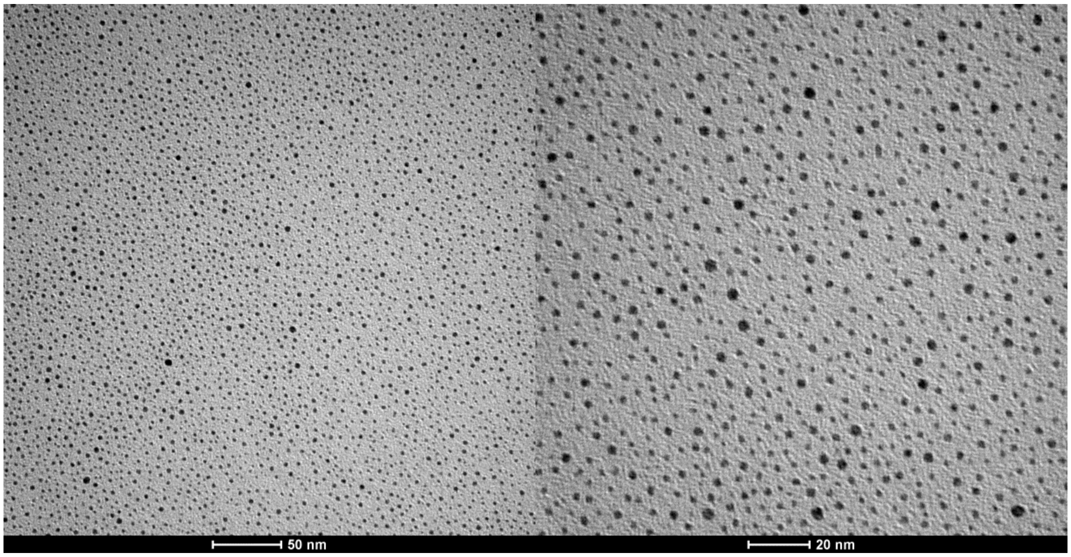

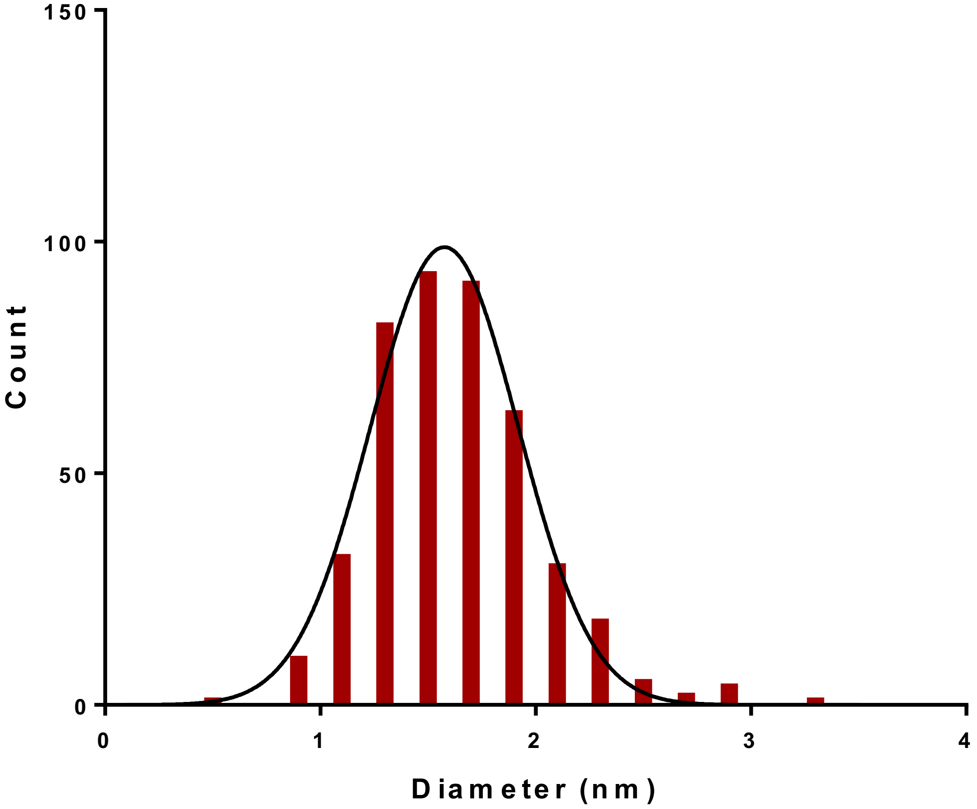

Figure S8. Sample TEM images **ZW-NP** (top) and size distribution (bottom): average diameter 1.6 nm (σ=0.3 nm).

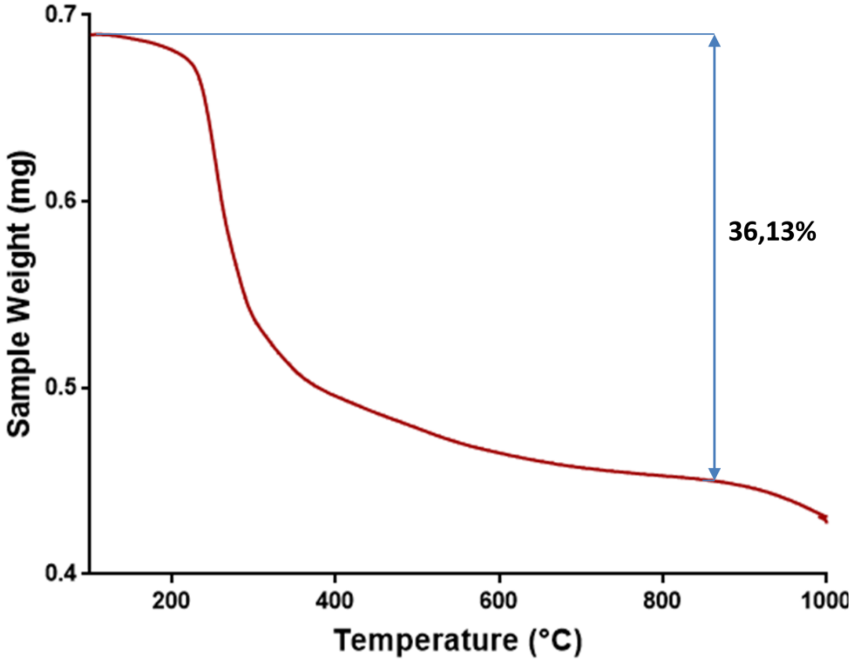

Figure S9. TGA analysis of **ZW-NP** (0.68 mg, 100 °C to 1000 °C, ramp 10 °C/min, air atmosphere). Weight loss 36.13%.

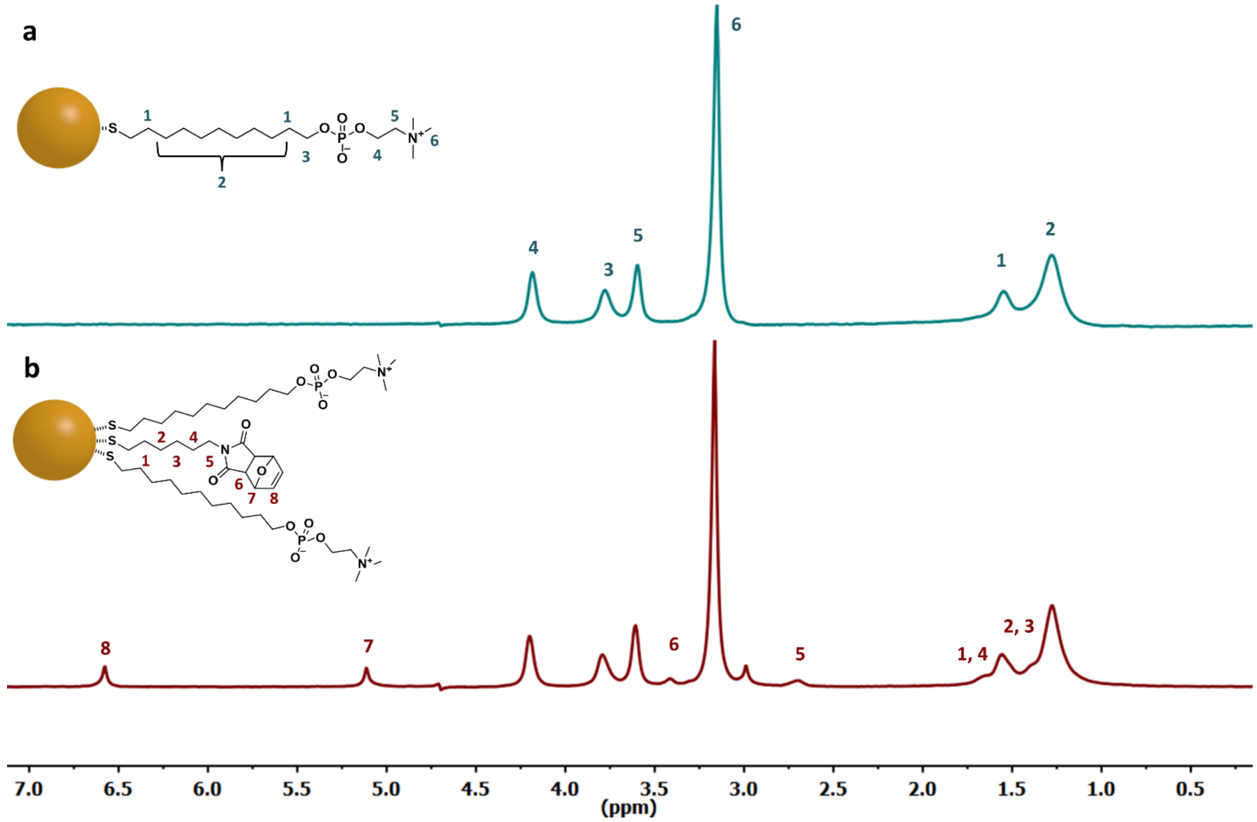

Figure S10. (**a**) Diffusion filter spectrum in D_2O of **ZW-NP** (30 µM, 2 mM in thiols) with assignation of the monolayer resonances. Only the signals of the molecules attached to the gold core appear on this spectrum. **(b)** Diffusion filter spectrum in D_2O of **NP-1** (30 µM, 2 mM in thiols) that confirms that after the thiol exchange reaction both **ZW-SH** and **MalDA-SH** are present in the coating monolayer.

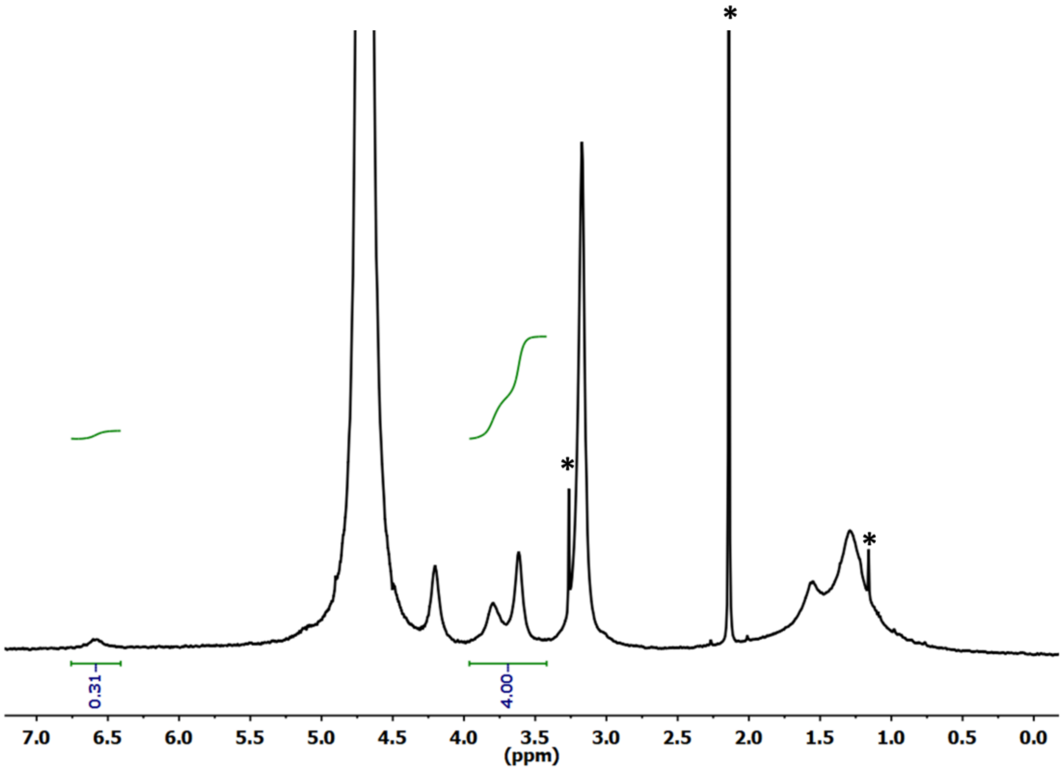

Figure S11. Evaluation of the percentage of **MalDA-SH** present on **NP-1**, from the ^{1}H-NMR spectrum in D_2O of **NP-1**. The integral of a signal of **ZW-SH** (4H, 3.70 ppm) was compared to the integral of a signal of **MalDA-SH**. Ratio **ZW-SH : MalDA-SH** 86.2:13.8 Asterisks denote solvent impurities.

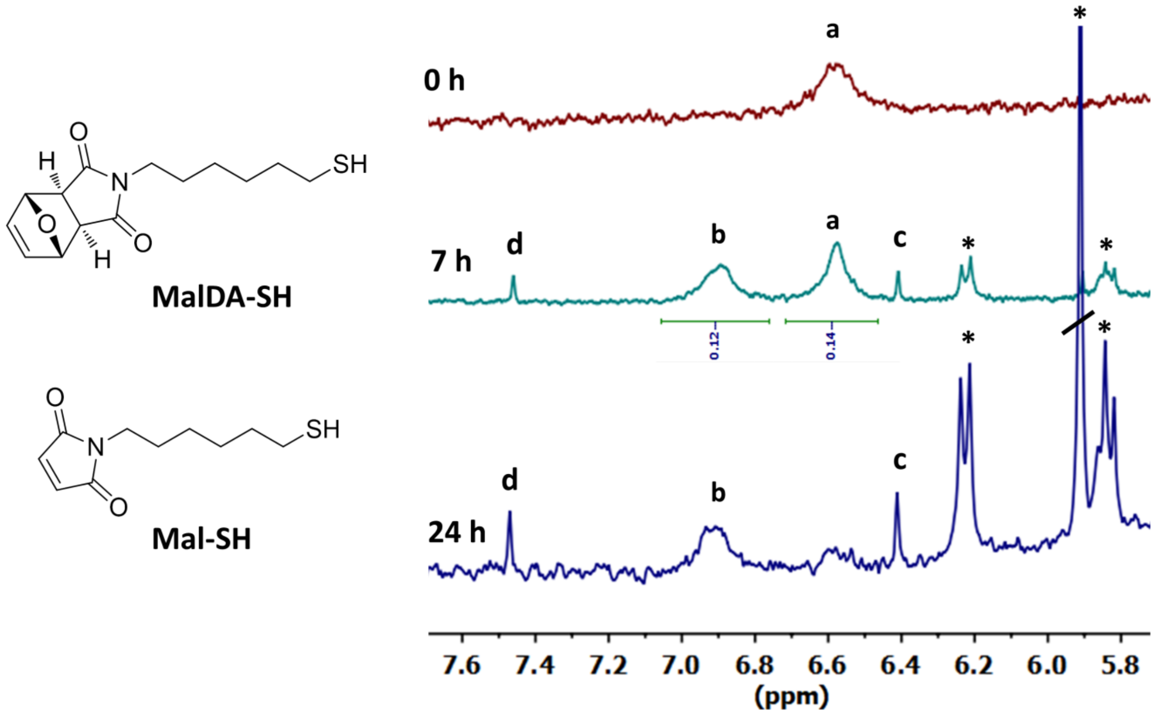

Figure S12. Comparison of a region of the ^{1}H-NMR spectra of **NP-1** (15 μM, 1 mM in thiol) and of the same sample after 7 h at 90°C. The signal *a* of the **MalDA-SH** is decreasing and new signals appear. Signal *b* can be assigned to the product of deprotected **Mal-SH**, signal *c* to furan and the signals marked with the asterisks denote unknown degradation products. Comparing, as in figure S4, the integrals of the peaks *a* and *b*, in the 7 h spectrum, with the **ZW-SH** peaks at 3.70 ppm (not shown), it appears that after seven hours of deprotection on the nanoparticle are present 6.5% of **MalDA-SH** and 5.6% of **Mal-SH**. After 24h the deprotection is almost completed, but the amount of degradation products is increasing. The intensity of the singlet at 5.9 ppm has been reduced for clarity.

5 Supplementary references

Chouikhi, D., Ciobanu, M., Zambaldo, C., Duplan, V., Barluenga, S., and Winssinger, N. (2012). Expanding the Scope of PNA-Encoded Synthesis (PES): Mtt-Protected PNA Fully Orthogonal to Fmoc Chemistry and a Broad Array of Robust Diversity-Generating Reactions. *Chem. - A Eur. J.* 18, 12698–12704. doi:10.1002/chem.201201337.

Holmlin, R. E., Chen, X., Chapman, R. G., Takayama, S., and Whitesides, G. M. (2001). Zwitterionic SAMs that resist nonspecific adsorption of protein from aqueous buffer. *Langmuir* 17, 2841–2850. doi:10.1021/la0015258.

Huang, Z., Zhao, J., Wang, Z., Meng, F., Ding, K., Pan, X., et al. (2017). Combining Orthogonal Chain-End Deprotections and Thiol-Maleimide Michael Coupling: Engineering Discrete Oligomers by an Iterative Growth Strategy. *Angew. Chemie Int. Ed.* 56, 13612–13617. doi:10.1002/anie.201706522.

Manea, F., Bindoli, C., Polizzi, S., Lay, L., and Scrimin, P. (2008). Expeditious synthesis of water-soluble, monolayer-protected gold nanoparticles of controlled size and monolayer composition. *Langmuir* 24, 4120–4124. doi:10.1021/la703558y.
